# The influence of front‐of‐pack portion size images on children's serving and intake of cereal

**DOI:** 10.1111/ijpo.12583

**Published:** 2019-11-20

**Authors:** Lauren Sophie McGale, Tim Smits, Jason Christian Grovenor Halford, Joanne Alison Harrold, Emma Jane Boyland

**Affiliations:** ^1^ Department of Psychological Sciences University of Liverpool Liverpool UK; ^2^ Institute for Media Studies Katholieke Universiteit Leuven Leuven Belgium

**Keywords:** Appetite, childhood obesity, marketing, portion size

## Abstract

**Background:**

Consumption of large portions of energy‐dense foods promotes weight gain in children. Breakfast cereal boxes often show portions much larger than the recommended serving size.

**Objective:**

This experimental study investigated whether front‐of‐package portion size depictions influence children's self‐served portions and consumption.

**Methods:**

In a between‐subjects design, 41 children aged 7‐11 years (M= 9.0 ± 1.5y) served themselves breakfast cereal from a box, the front of which depicted either a recommended serving size of cereal (30g) or a larger, more typical front‐of‐pack portion (90g). Cereal served and consumed and total caloric intake (including milk) was recorded. Height and weight, demographic information and measures of children's food responsiveness and enjoyment of food were collected.

**Results:**

MANOVA revealed that children exposed to the larger portion size served themselves (+7g, 37%) and consumed (+6g, 63%) significantly more cereal than those exposed to the smaller portion. Despite this, overall caloric intake (milk included) did not differ between conditions, and no other measured variables (hunger, BMI) significantly affected the outcomes.

**Conclusion:**

This study provides novel evidence of the influence portion‐size depictions on food packaging have on children's eating behaviour. This offers possible avenues for intervention and policy change; however, more research is needed.

**Abbreviations**
M: mean
y: year
g: grams
BMI: Body Mass Index
US: United States
UK: United Kingdom
CEBQ: Child Eating Behaviour Questionnaire
VAS: visual analogue scale
g: grams
FR: Food Responsiveness
EF: Enjoyment of Food
WHO: World Health Organization
kg/m^2^: kilograms per metre squared
MANOVA: Multivariate Analysis of Variance
MANCOVA: Multivariate Analysis of Covariance
Kcals: kilocalories

## 1 INTRODUCTION

Across Europe and the US, portion sizes have been increasing in parallel with increases in body weight.^1^, ^2^, ^3^, ^4^ This could reflect a number of phenomena, including marketers' responses to customer preferences for larger portions or greater value (with larger portions per unit reducing production costs), and it could be argued that marketers may actually be shaping these preferences too – leveraging the portion‐size effect in order to increase consumption and further purchasing.^5^

A meta‐analytic review found that portion size had a significant effect (medium‐sized, d = .45) on consumption.^5^ Specifically, when a portion size was doubled, consumption increased by an average of 35%, across a range of contexts and foods, in both adults and children. In children, larger portion sizes have consistently been shown to result in increased consumption in both the laboratory^6^, ^7^, ^8^, ^9^ and naturalistic settings, such as childcare centres and preschool/school canteens.^8^, ^10^, ^11^, ^12^, ^13^

Consumption norms (perceptual suggestions of what is appropriate, typical and reasonable to consume) have influence outside of conscious awareness^14^ and have been suggested as a driver of this ‘portion size effect',^5^ with portion sizes communicating normative information regarding appropriate consumption.^15^ Furthermore, perceptions of what constitutes an appropriate portion size have been shown to partially mediate the relationship between portion size and food served,^15^ and to predict food consumption.^16^

Tentative evidence suggests that these perceptions of appropriate portion sizes may be malleable, with people normalising the portion sizes they are exposed to, and so may result in larger portion sizes becoming ‘normal'. A recent experimental study found that mere visual exposure to a larger or smaller portion of a snack food, without consumption, affected intake of that food twenty‐four hours later.^17^ Nevertheless, there is a scarcity of evidence to date which explores the impact of visual exposure to a portion cue on eating behaviour, specifically in children.

Depictions of portion sizes (often referred to as ‘serving suggestions') are frequently used on the front of food packaging as part of efforts to present foods in a visually appealing, salient manner. These images may offer a normative reference point for consumers through repeated exposure, providing an implicit cue which suggests that this image shows what an appropriate portion should look like. However, studies have shown that the image typically represents a much larger portion size than the recommended serving (usually stated on the side/back of packaging). A study of 158 cereal boxes in the US found that portion size depictions were, on average, 64.7% larger than the recommended portion (221 vs 134 calories).^18^ A subsequent experimental study in a student population found that cereal boxes depicting inflated portion sizes led participants to serve themselves 17.8% more cereal compared with boxes that showed a more realistic (recommended) portion size.^18^ However, the students' actual consumption of the cereal was not measured.

Cereal is frequently marketed to children, in particular, high sugar/low fibre, ready‐to‐eat breakfast cereals,^19^, ^20^ and is not only a popular breakfast item amongst UK children but is also regularly consumed between meals as a snack.^21^ The promotion of these cereals is therefore of particular interest from a public health perspective, as a diet high in sugar and/or low in fibre will not only have implications regarding weight gain, but may lead to nutritional imbalances and additional health problems, independently of overweight, such as dental cavities or type 2 diabetes, for example.^22^, ^23^, ^24^ Although the impact of food commercials on children's food consumption is well documented,^25^ there is a relative paucity of evidence addressing the influence of more subtle marketing techniques (such as front‐of‐pack serving suggestions) on children's eating behaviours. As visual cues are the first sensory stimuli of an eating experience, they are thought to play a key role in influencing consumption and choice.^26^ Boswell & Kober's^27^ meta‐analysis found that visual food cues (such as images of food), when combined with experience of craving, were significantly associated with eating behaviour with a similar effect size to real food exposure and a stronger effect size than olfactory cues. Based on existing research, it is plausible to predict that inflated visual portion size cues presented on food packaging may affect food‐related outcomes such as intake. Given the sugar content of these cereals (with some found to be more than 1/3 sugar by weight^21^), the potential implications of inflated portion sizes on children's dietary health are clear.

An experimental study by Neyens, Aerts, & Smits^28^ sought to explore this phenomenon by manipulating the images of food presented on a novel children's cereal. The size of the image used on the front‐of‐pack was adjusted, but the portion size shown within the images was held constant. Children exposed to the larger sized image both served and consumed more cereal and milk than those who were exposed to a smaller sized image. This suggests that the provision of a reference amount for serving and intake may inform children's decisions by signalling an appropriate amount to eat (consumption norm). The current study sought to explore this effect further, by manipulating the actual portion size depicted within the images, rather than the image size itself using a between‐subjects experiment with two portion size depiction conditions.

The study hypotheses were as follows. Primary hypotheses: (i) children will serve themselves more cereal in the normal (large) portion size condition; (ii) children will consume more cereal in the normal (large) portion size condition; (iii) children will both serve and consume more overall (cereal and milk) in the normal (large) portion size condition. Secondary hypotheses: (iv) children would accept the portion‐size depicted as appropriate, regardless of condition.

## 2 METHODS

Neyens et al.^28^ found a large effect size of 0.9 using a within‐subjects design. To be conservative, the current study was powered for a medium‐large effect size (d = 0.6, 95% power, *P* < .05) and with a between‐subjects design, power analyses (G*Power software v3.1) showed that a sample size of 39 was needed. However, as this was an opportunity sample, 41 children aged 7‐11 years (9.0 ± 1.5y; 21 female, 51.2%) were recruited from five school breakfast clubs and one childcare centre in the UK.

The study was approved by the University of Liverpool's Ethics Sub‐committee for Non‐invasive Procedures. Head teachers, childcare centre directors, and parents provided informed, written consent. Parents also supplied demographic and lifestyle information (gender, age, parental education, ethnicity, whether children typically consumed cereal for breakfast and if they typically served themselves) and completed two subscales of the CEBQ,^29^ pertaining to food responsiveness (FR) and enjoyment of food (EF) [discussed in supplementary materials]. Participating children gave verbal assent for participation and data were collected between February and November 2015. Child demographics are reported in Table 1.

**Table 1:** Table detailing demographic characteristics of children

*Demographics*	
Age, mean ± SD (range), y	9.0 ± 1.5y (5.3‐11.9y)
Gender, *n (*%)	
Male	22 (53.7)
Female	19 (46.3)
BMI, Mean ± SD (range)	17.1 ± 2.8kg/m^2^ (12.5 – 23.9kg/m^2^)
Weight Status, *n* (%)	
NW	34 (82.9)
OWOB	7 (17.1)

*Note*. BMI, body mass index; NW, normal weight; OWOB, overweight or obese; SD, standard deviation; y, year.

**Figure 1:**
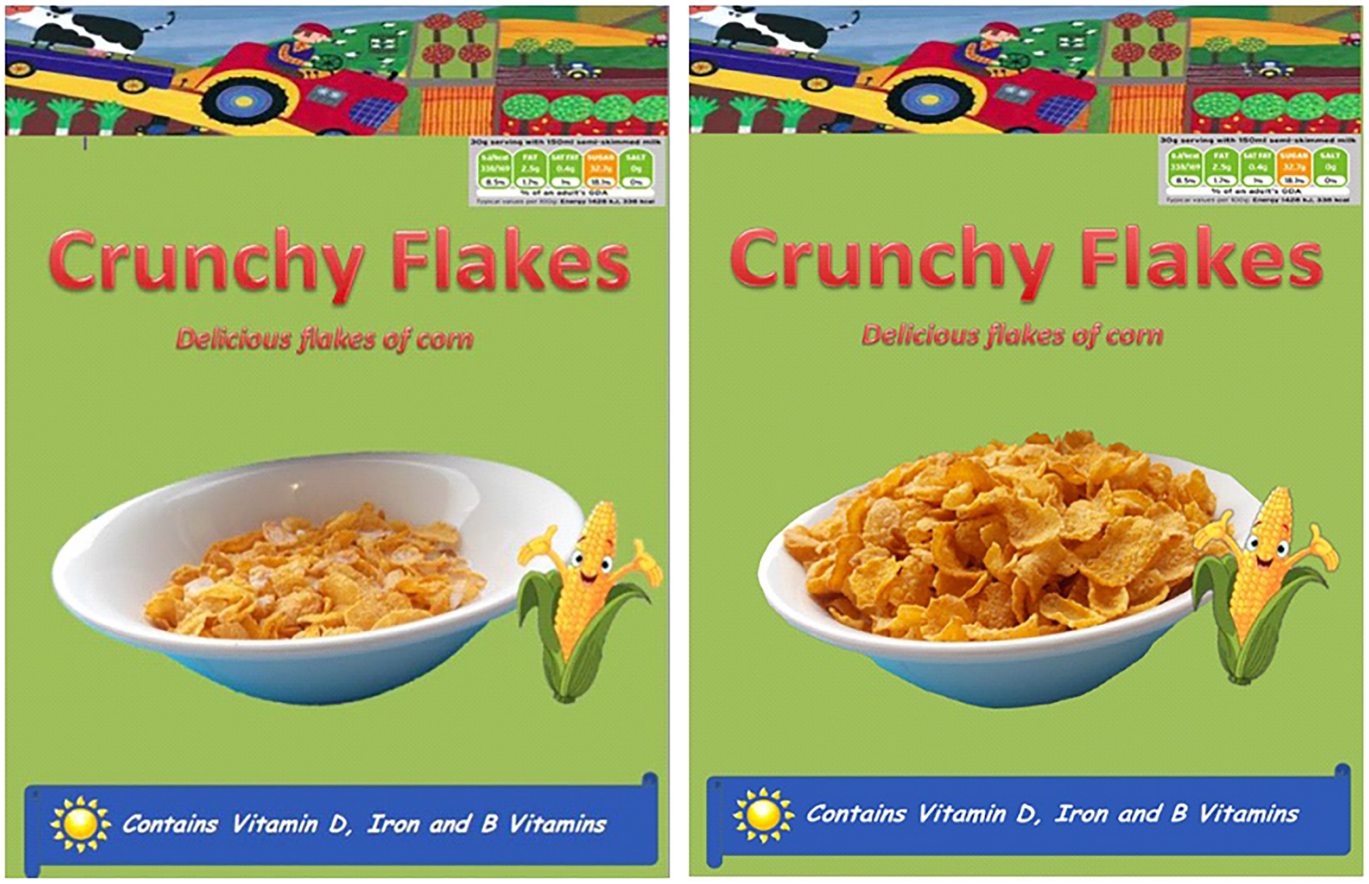
Cereal boxes for small and large portion size depiction conditions, respectively.

The study used a between‐subjects design, with two portion‐size conditions: children were exposed to a cereal box depicting (i) a small visual cue (the image showed an amount of cereal in the bowl that was consistent with the written gram serving suggestion stated on the pack; 30g) or (ii) a normal (large) visual cue (the bowl contained a larger portion containing three times the recommended serving, representing a normal visual cue, as commonly found on cereal packaging; 90g). Two novel cereal packages were designed for this study (using CorelDRAW X7), differing only by the portion size depicted on the front‐of‐pack (see Figure 1). The boxes were designed with typical, commercially available cereal boxes in mind and the cereal used was Kellogg's Corn Flakes®. The bowls used, as well as the weight of the cereal boxes and milk served, were kept consistent for all participants.

Children were tested individually. After being given an age‐appropriate explanation, verbal assent was obtained. Only when children confirmed they had not eaten breakfast were they included and assigned to an experimental condition using a simple randomisation schedule (http://www.randomizer.org). In accordance with this schedule, children were presented with a cereal box with either the small portion size or normal portion size (large) depicted on the front. Full cereal boxes were always presented and were pre‐weighed each time to ensure accurate intake measurements could be obtained. With the cereal box present, children were given a series of child‐friendly ‘smiley face' visual analogue scales (VAS) to rate hunger, expectation of cereal liking, whether they felt the portion depicted was appropriate or not and opinions on various aspects of the cereal packaging (dummy questions were included to ensure children gave attention to the manipulated imagery). Children were then given a bowl, a spoon and a standardised serving of semi‐skimmed milk from which to pour (the UK equivalent of 2% milk; 100g). They were instructed to serve themselves their breakfast (cereal and milk), and advised that they would be asked to give their opinions on how the cereal tasted once they finished their meal; children were not given a time limit in which to complete their meal. Once children indicated that they had finished, cereal boxes, milk and bowls were removed and weighed discreetly in order to ascertain the amount served and consumed (grams). Post‐meal VAS were administered to assess hunger, liking of cereal and packaging. Measures of height and weight were taken individually in private and an age appropriate debrief was given to each child, along with a debrief letter for parent(s)/guardian(s).

For use in analyses, Body Mass Index (BMI) was converted to an age‐ and gender‐appropriate z‐score, using the World Health Organization Anthropometric Calculator software (WHO Anthro version 3.2.2.) and weight status was subsequently defined using age‐ and gender‐specific BMI cut‐off points, which are equivalent to adult BMIs of 25 kg/m^2^ (overweight) and 30 kg/m^2^ (obese), as recommended by the World Obesity Federation.^30^

Outcome data were checked to ensure assumptions for parametric data were met. Data for milk consumed (grams) and milk served (grams) were found to be skewed, with moderate‐low skewness of ‐.08 (*SE* = .05) and kurtosis of ‐1.16 (SE = .97) for milk consumed (grams), and moderate‐high skewness of ‐.94 (*SE* = .05) and kurtosis of ‐.48 (SE = .97) for milk served (grams). A Shapiro‐Wilk test revealed that normality can be assumed for the milk consumed (W= .91, *P* = .07), however, data for milk served is not normally distributed ((W= .75, *P* = .000). As MANOVA it is generally considered to be robust enough to cope with small deviations such as this, it was still included in the model.

All comparisons were two‐tailed and significance was taken as *P* < .05 (with Bonferroni adjustments for multiple comparisons). Where assumptions of sphericity were violated, a Greenhouse‐Geisser correction was used. Analyses were completed using SPSS v24 for Windows (SPSS Inc., Chicago, US).

## 3 RESULTS

Participants did not differ significantly between conditions on any of the following variables: age, gender, BMI, pre‐meal hunger, pre‐meal perceived liking of cereal, post‐meal ratings of actual cereal liking, habitual breakfast eating and habitual breakfast self‐serving (*P* > .05). Of the 41 participants, 37 (90%) returned a parental questionnaire (see Table 2).

**Table 2:** Demographic and lifestyle characteristics of participants (as a % of completed parental questionnaires)

***Parental questionnaire***	
Completed, *n* (%)	37 (90)
Mother	29 (70.7)
Father	5 (12.2)
*Undisclosed*	*7 (17.1)*
Ethnicity, *n* (%)	
British ‐ White	28 (68.3)
British ‐ Other	4 (9.7)
Mixed ‐ Other	1 (2.4)
*Undisclosed*	*7 (17.1)*
Parental education level (%)	
Post‐graduate	0 (0)
Degree	3 (7.3)
A levels	15 (36.6)
GCSE	6 (14.6)
Other	8 (19.5)
*Undisclosed*	*9 (22)*
Typically eat cereal for breakfast, *n* (%)	
Yes	29 (70.7)
No	8 (19.5)
*Undisclosed*	*4 (9.8)*
Typically serve themselves, *n* (%)	
Yes	14 (34.1)
No	23 (56.1)
*Undisclosed*	*4 (9.8)*
CEBQ scores, *Mean* (SD)	
Food responsiveness	12.1 (5.15)
Emotional over‐eating	7.08 (2.29)
Enjoyment of food	9.11 (1.67)
Desire to drink	8.86 (3.67)
Satiety responsiveness	14.39 (3.97)
Slowness in eating	11.31 (3.81)
Emotional under‐eating	9.61 (3.68)
Food fussiness	15.17 (3.01)

*Note*. A levels: General Certificate of Education Advanced level (UK); CEBQ, Children's Eating Behaviour Questionnaire; GCSE, General Certificate in Secondary Education (UK); SD, standard deviation.

### 3.1 Primary Results

A one‐way multivariate analysis of variance (MANOVA) was run to determine the effect of condition (normal vs small portion depiction) on the three primary outcome variables (in grams): i) cereal serving, ii) cereal consumption and iii) total meal (cereal and milk) consumption.

Overall, the model was statistically significant, indicating differences between the two portion size conditions on the combined eating behaviour variables (*F*(1,38) = 5.61, *P* = .015, Wilk's Λ = 0.68, partial η2 = .32). A significant main effect of condition (normal vs small portion depiction) was found for weight of cereal served (*F*(1,38) = 6.55, *P* = .015, partial η2 = .15) and cereal consumed (*F*(1,38) = 10.901, *P* = .002, partial η2 = .22). Children in the normal portion conditions both served and consumed more cereal than those shown the small portion image. A significant main effect of condition on the total weight of the meal (cereal and milk) consumed was also found (*F*(1,38) = 6.02, *P* = .019, partial η2 = .14; see Table 3 for means), however, this main effect disappears when covariates were introduced to the model. The overall model was not influenced by BMI z‐scores, age, sex, pre‐meal ratings of hunger or pre‐meal ratings of expected liking of cereal when these factors were included as covariates (*P = .023*). Multivariate analysis of covariance (MANCOVA) results are reported in full in supplementary materials.

**Table 3:** Table detailing mean weight (grams) of outcome variables (mean ± SE), by condition

Outcome Variable	**Portion size condition**	
	*Small*	*Normal (large)*
Cereal served*	18.68 ± 1.94	25.53 ± 1.85
Cereal consumed**	10.07 ± 1.40	16.43 ± 1.33
Total meal consumed^a^	47.10 ± 7.77	73.41 ± 7.39

Note.

* *P* < .05,

** *P* < .01. ^a^One child was given 69.4g of milk in error (instead of 100g). Sensitivity analyses showed that removing this participant did not affect the outcomes and so these data were retained in the final model. SE, standard error.

### 3.2 Secondary Results

Overall, 63% of children accepted the image on the cereal box as accurately depicting an appropriate portion size, with 20% believing there was not enough cereal in the bowl and 17% believing there was too much. A one‐way analysis of variance showed no significant main effect of condition (*F*(1,39) = 3.4, *P* = .07); serving norms did not differ between conditions, indicating children's acceptance of the image as depicting an appropriate portion, regardless of whether they were shown the normal or small portion. See Table 4 for breakdown of percentages by condition.

**Table 4:** Table detailing children's perceptions of the portion size image depicted, by condition; *n* (%)

Response	**Portion size condition**	
	*Small*	*Normal (large)*
Not enough cereal	7 (35)	1 (4.8)
The right amount of cereal	10 (50)	16 (76.2)
Too much cereal	3 (15)	4 (19)

See supplementary materials for the results of a series of two way MANOVAs determining whether an interaction exists between condition and the relevant CEBQ scales (food responsiveness [FR] and enjoyment of food [EF]) on the outcome variables (cereal served, cereal consumed and total meal consumed [grams]).

## 4 DISCUSSION

To our knowledge, this is the first study to empirically demonstrate that altering the portion size shown in food images depicted on food packaging influences the serving and consumption behaviour of children. It provides evidence for a main effect of portion size depictions on both children's serving sizes and consumption, with the normal (large) portion condition resulting in children serving and consuming significantly more cereal than those who were shown a smaller portion size image. Furthermore, it provides evidence that children may accept the serving suggestions on food packaging as an indication of an appropriate portion size, even when the image represents three times the true recommended serving (90g of cereal).

This supports a wealth of existing empirical support for the portion size effect, but also for the effect of external visual cues on children's food behaviours. For example, a meta‐analysis by Boswell and Kober^27^ found that visual cues, such as videos and pictures, were associated with medium‐sized effects on eating and weight, similar to real food cues. Additionally, it lends direct support to a growing body of research which suggests children's eating behaviours are influenced by visual cues of portion‐size on food packaging. Neyens, Aerts and Smits,^28^ found that adjusting the size of the serving suggestion image influenced serving and consumption in 4‐5 year old children. More recently, Aerts and Smits,^31^ across two studies, demonstrated that children consumed more food when it was presented in packaging which depicted a larger portion size, when compared with a normal one.

Behavioural research has shown that using peers as indicators of consumption norms could influence children's eating behaviour.^32^, ^33^ It is suggested that children will conform to perceived consumption norms,^34^ and normative benchmarks are influential enough to be impactful even when indicated by way of a remote/fictitious confederate.^35^ Portion size depictions on food packaging, as a consumption norm indicator, are more covert than the obvious and observable behaviour of a peer. However, they may subtly portray a ‘normal' serving and remove children's uncertainty about how to behave in novel contexts, for example, serving food, which is typically done by a caregiver.^34^

It would be reasonable to expect that, through repeated exposure and habituation, the imagery used in the normal (large) portion condition, showing 90g of cereal, which is consistent with recent evidence on typical real‐world food packaging,^21^ would be perceived by the children as normal, and the smaller image as not normal. However, the majority of children accepted the portion depicted as appropriate, regardless of condition, lending further support to the notion that children are vulnerable to manipulations of external cues. Furthermore, this supports recent adult literature which found that mere visual exposure to portion sizes influenced perceptions of portion size normality and subsequent consumption.^17^ Future research should explore norm ranges and the effects of a wider range of portion sizes on intake in children.

Research has demonstrated that a positive energy gap of as little as 69‐77kcal per day was responsible for weight gain or weight maintenance in children who were already overweight,^36^ and Plachta‐Danielzik et al.^37^ recommend that, in order to prevent overweight in children, excess energy should not exceed 46‐72kcal per day. The increase in consumption in the current study, with ~7 grams more cereal being consumed in the normal (large) portion condition, equates to an increase of ~25kcal. This constitutes half of the daily excess energy requirement for the development of overweight in children. As this represents only one of several meals they will consume in a day, there are further opportunities for excess calories to be consumed throughout the day, increasing the likelihood of overweight development. Furthermore, the cereal used in this study (cornflakes) does not contain as much sugar as many cereals aimed at children (8g/100g), so these results may underestimate the true caloric impact of this effect.

Cornflakes are a plain breakfast cereal, both visually and in taste, and so may not have appealed strongly to the children; as such, this approach was likely to result in a more conservative effect on eating behaviours. Future studies may wish to use more visually appealing foods, such as those typically marketed to children, in order to establish any potential differences in effects.

A further consideration when interpreting results is the order of procedures. Children were prompted to look at the cereal box imagery before serving and consuming the cereal. This was to ensure children noted and engaged with the manipulation, but may have also acted as a prime. Future studies could ask questions regarding the packaging post‐meal to allow this effect to be disentangled from that of the image manipulation itself.

The current study used a natural control, in the form of the large portion size condition, which is typically representative of cereal packaging currently on the market. Future studies may wish to include an additional “no portion image” control condition, to establish how children respond in the absence of a visual cue. However, the current study design was pragmatic, seeking to reflect a more realistic policy option (a requirement for manufacturers to use front‐of‐pack imagery that accurately indicates the recommended portion size) than total removal of product images from packaging

Children with obesity have been shown to be less responsive to internal satiety cues, and more sensitive to external food cues, than children of a healthy weight in many^38^, ^39^ but not all studies.^33^ Imbalances in the group sizes in the current study meant statistical analyses involving weight status categories were not appropriate. Future studies should seek to recruit participants equally across weight status categories to allow for this.

This study has some limitations. The majority of children served themselves all of the milk provided, rather than an amount proportionate to their cereal serving resulting in negatively skewed data. This may have created a ceiling effect, and could explain why the findings for the total meal consumed were not consistent with the effects found for the primary outcomes, cereal serving and intake. It is likely that children, when given the option to serve milk *ad libitum* in the home*,* would serve more milk with more cereal. Future studies should therefore provide larger portions of milk from which to serve, allowing for more variation within the data.

The current study is a conceptual replication and an extension of an existing body of literature which suggests visual cues on food packaging influence children's eating behaviours.^28^, ^31^ However, as the first study to measure this particular phenomenon, findings are to be approached with caution. Certainly direct replication of the findings is required before firm conclusions can be drawn. Furthermore, replications with differing stimuli such as a variety of foods or more palatable breakfast cereals, for example, would strengthen the claim.

It has yet to be established whether or not manipulating front‐of‐pack depictions of food portions would have any influence over adolescents or adults. It is reasonable to assume that due to habitual serving and consumption norms developed over years, visual cues would be less likely to affect older populations, however, this is conjecture and empirical evidence is required to ensure that any interventions can be applied to the relevant populations. Due to the habitual nature of cereal serving, future research may wish to evaluate this manipulation in relation to more novel foods, for example, foods which participants do not recognise or report consuming with less frequency. Nevertheless, this study has been the first to address this particular phenomenon, demonstrating that when large portion sizes of cereal are depicted on the front of cereal packaging children serve themselves and consume more cereal. From a public health and food policy perspective, these findings sit within a body of research that, if replicated and extended as discussed, could have implications for policy and regulations which govern food packaging and front‐of‐pack marketing to children.

## 5 CONCLUSION

Exposure to visual cues such as portion size depictions on food packaging influences children's self‐serving and consumption behaviours. The findings presented here could potentially contribute to public health strategies for obesity reduction and policy deliberations around the marketing of foods to children.

### CONFLICT OF INTEREST STATEMENT

JCGH and JAH have received funding to their institution from the American Beverage Association. The other authors have no conflicts of interest relevant to this article to disclose. No external funding was received for this research.

## Supporting information

Table S1. Results of one‐way MANCOVA showing the effects of condition (large vs small portion depiction) on three primary outcome variables (cereal serving, cereal consumption and total meal consumption), controlling for BMI, age, sex, pre‐meal ratings of liking and pre‐meal ratings of hunger.
Table S2. Results of two‐way MANCOVA showing interaction effects between condition (large vs small portion depiction) and food responsiveness on the three primary outcome variables (cereal serving, cereal consumption and total meal consumption)
Table S3. Results of two‐way MANCOVA showing interaction effects between condition (large vs small portion depiction) and enjoyment of food on the three primary outcome variables (cereal serving, cereal consumption and total meal consumption)

## References

[1] Matthiessen J , Fagt S , Biltoft‐Jensen A , Beck AM , Ovesen L . Size makes a difference. Public Health Nutr. 2003;6(1):65‐72. 10.1079/PHN2002361 12581467

[2] Nielsen SJ , Popkin BM . Patterns and trends in food portion sizes, 1977‐1998. JAMA. 2003;289(4):450‐453. http://www.ncbi.nlm.nih.gov/pubmed/12533124 12533124 10.1001/jama.289.4.450

[3] Piernas C , Popkin BM . Food portion patterns and trends among U.S. children and the relationship to total eating occasion size, 1977‐2006. J Nutr. 2011;141(6):1159‐1164. 10.3945/jn.111.138727 21525258 PMC3095143

[4] Young LR , Nestle M . The contribution of expanding portion sizes to the US obesity epidemic. Am J Public Health. 2002;92(2):246‐249. http://www.ncbi.nlm.nih.gov/pubmed/11818300 11818300 10.2105/ajph.92.2.246 PMC1447051

[5] Zlatevska N , Dubelaar C , Holden SS . Sizing Up the Effect of Portion Size on Consumption: A Meta‐Analytic Review. https://doi.org/101509/jm120303. 2014.

[6] Fisher JO , Liu Y , Birch LL , Rolls BJ . Effects of portion size and energy density on young children's intake at a meal. Am J Clin Nutr. 2007;86(1):174‐179. http://www.ncbi.nlm.nih.gov/pubmed/17616778 17616778 10.1093/ajcn/86.1.174 PMC2531150

[7] Kral TVE , Kabay AC , Roe LS , Rolls BJ . Effects of doubling the portion size of fruit and vegetable side dishes on children's intake at a meal. Obesity (Silver Spring). 2010;18(3):521‐527. 10.1038/oby.2009.243 19680238

[8] Looney SM , Raynor HA . Impact of Portion Size and Energy Density on Snack Intake in Preschool‐Aged Children. J Am Diet Assoc. 2011;111(3):414‐418. 10.1016/j.jada.2010.11.016 21338741

[9] Mathias KC , Rolls BJ , Birch LL , et al. Serving larger portions of fruits and vegetables together at dinner promotes intake of both foods among young children. J Acad Nutr Diet. 2012;112(2):266‐270. http://www.ncbi.nlm.nih.gov/pubmed/22741168 22741168 10.1016/j.jada.2011.08.040 PMC3776004

[10] Huss LR , Laurentz S , Fisher JO , McCabe GP , Kranz S . Timing of serving dessert but not portion size affects young children's intake at lunchtime. Appetite. 2013;68:158‐163. 10.1016/j.appet.2013.04.013 23619315

[11] Kling SMR , Roe LS , Keller KL , Rolls BJ . Double trouble: Portion size and energy density combine to increase preschool children's lunch intake. Physiol Behav. 2016;162:18‐26. 10.1016/j.physbeh.2016.02.019 26879105 PMC4899121

[12] Smith L , Conroy K , Wen H , Rui L , Humphries D . Portion size variably affects food intake of 6‐year‐old and 4‐year‐old children in Kunming. China Appetite. 2013;69:31‐38. 10.1016/j.appet.2013.05.010 23702260 PMC4319705

[13] Aerts G , Smits T . The package size effect: How package size affects young children's consumption of snacks differing in sweetness. Food Qual Prefer. 2017;60:72‐80. 10.1016/J.FOODQUAL.2017.03.015

[14] Wansink B , van Ittersum K . Portion size me: downsizing our consumption norms. J Am Diet Assoc. 2007;107(7):1103‐1106. 10.1016/j.jada.2007.05.019 17604738

[15] Versluis I , Papies EK . The Role of Social Norms in the Portion Size Effect: Reducing Normative Relevance Reduces the Effect of Portion Size on Consumption Decisions. Front Psychol. 2016;7:756 10.3389/fpsyg.2016.00756 27303324 PMC4885850

[16] Haynes A , Hardman CA , Makin ADJ , Halford JCG , Jebb SA , Robinson E . Visual perceptions of portion size normality and intended food consumption: A norm range model. Food Qual Prefer. 2018;72:77‐85. 10.1016/j.foodqual.2018.10.003 PMC6333281 30828136

[17] Robinson E , Henderson J , Gregory KS , Kersbergen I . When a portion becomes a norm: Exposure to a smaller vs. larger portion of food affects later food intake. Food Qual Prefer. February 2019 10.1016/J.FOODQUAL.2019.02.013 PMC7086457 32226235

[18] Tal A , Niemann S , Wansink B . Depicted serving size: cereal packaging pictures exaggerate serving sizes and promote overserving. BMC Public Health. 2017;17(1):169‐167. 10.1186/s12889-017-4082-5 28166756 PMC5294869

[19] LoDolce ME , Harris JL , Schwartz MB . Sugar as Part of a Balanced Breakfast? What Cereal Advertisements Teach Children About Healthy Eating. J Health Commun. 2013;18(11):1293‐1309. 10.1080/10810730.2013.778366 24175878

[20] Whalen R , Harrold J , Child S , Halford J , Boyland E . Children's exposure to food advertising: the impact of statutory restrictions. Health Promot Int. October 2017 10.1093/heapro/dax044 29092014

[21] Khehra R , Fairchild RM , Morgan MZ . UK children's breakfast cereals – an oral health perspective. BDJ. 2018;225(2):164‐169. 10.1038/sj.bdj.2018.531 30050223

[22] Sonestedt E , Overby NC , Laaksonen DE , Birgisdottir BE . Does high sugar consumption exacerbate cardiometabolic risk factors and increase the risk of type 2 diabetes and cardiovascular disease? Food Nutr Res. 2012;56 10.3402/fnr.v56i0.19104 PMC3409338 22855643

[23] Schulze MB , Manson JE , Ludwig DS , et al. Sugar‐sweetened beverages, weight gain, and incidence of type 2 diabetes in young and middle‐aged women. JAMA. 2004;292(8):927‐934. 10.1001/jama.292.8.927 15328324

[24] Skafida V , Chambers S . Positive association between sugar consumption and dental decay prevalence independent of oral hygiene in pre‐school children: a longitudinal prospective study. J Public Health (Oxf). 2018;40(3):e275‐e283. 10.1093/PUBMED/FDX184 29301042 PMC6166585

[25] Boyland EJ , Nolan S , Kelly B , et al. Advertising as a cue to consume: a systematic review and meta‐analysis of the effects of acute exposure to unhealthy food and nonalcoholic beverage advertising on intake in children and adults. Am J Clin Nutr. 2016;103(2):519‐533. 10.3945/ajcn.115.120022 26791177

[26] Reisfelt HH , Gabrielson G , Aaslyng MD , Bjerre MS , Møller P . Consumer preferences for visually presented meals. J Sens Stud. 2009;24(2):182‐203. 10.1111/j.1745-459X.2008.00202.x

[27] Boswell RG , Kober H . Food cue reactivity and craving predict eating and weight gain: a meta‐analytic review. Obes Rev. 2016;17(2):159‐177. 10.1111/obr.12354 26644270 PMC6042864

[28] Neyens E , Aerts G , Smits T . The impact of image‐size manipulation and sugar content on children's cereal consumption. Appetite. 2015;95:152‐157. 10.1016/j.appet.2015.07.003 26162951

[29] Wardle J , Guthrie CA , Sanderson S , Rapoport L . Development of the Children's Eating Behaviour Questionnaire. J Child Psychol Psychiatry. 2001;42(7):963‐970. 10.1111/1469-7610.00792 11693591

[30] Cole TJ , Bellizzi MC , Flegal KM , Dietz WH . Establishing a standard definition for child overweight and obesity worldwide: international survey. BMJ. 2000;320(7244):1240‐1243. http://www.ncbi.nlm.nih.gov/pubmed/10797032 10797032 10.1136/bmj.320.7244.1240 PMC27365

[31] Aerts G , Smits T . Do depicted suggestions of portion size on‐pack impact how much (un)healthy food children consume. Int J Consum Stud. 2019;43(3):237‐244. 10.1111/ijcs.12503

[32] Salvy S‐J , de la Haye K , Bowker JC , Hermans RCJ . Influence of peers and friends on children's and adolescents' eating and activity behaviors. Physiol Behav. 2012;106(3):369‐378. 10.1016/j.physbeh.2012.03.022 22480733 PMC3372499

[33] Romero ND , Epstein LH , Salvy S‐J . Peer Modeling Influences Girls' Snack Intake. J Am Diet Assoc. 2009;109(1):133‐136. 10.1016/j.jada.2008.10.005 19103334 PMC8078059

[34] Sharps M , Robinson E . Perceived eating norms and children's eating behaviour: An informational social influence account. Appetite. 2017;113:41‐50. 10.1016/J.APPET.2017.02.015 28192218 PMC5388191

[35] Bevelander KE , Anschütz DJ , Engels RCME . The effect of a fictitious peer on young children's choice of familiar v. unfamiliar low‐ and high‐energy‐dense foods. Br J Nutr. 2012;108(06):1126‐1133. 10.1017/S0007114511006374 22313605

[36] van den Berg SW , Boer JMA , Scholtens S , et al. Quantification of the energy gap in young overweight children. The PIAMA birth cohort study. BMC Public Health. 2011;11(1):326‐328. 10.1186/1471-2458-11-326 21586130 PMC3118240

[37] Plachta‐Danielzik S , Landsberg B , Bosy‐Westphal A , Johannsen M , Lange D , Müller MJ . Energy Gain and Energy Gap in Normal‐weight Children: Longitudinal Data of the KOPS. Obesity. 2008;16(4):777‐783. 10.1038/oby.2008.5 18379562

[38] Sleddens EF , Kremers SP , Thijs C . The Children's Eating Behaviour Questionnaire: factorial validity and association with Body Mass Index in Dutch children aged 6‐7. Int J Behav Nutr Phys Act. 2008;5(1):49 10.1186/1479-5868-5-49 18937832 PMC2612017

[39] Carnell S , Wardle J . Measuring behavioural susceptibility to obesity: Validation of the child eating behaviour questionnaire. Appetite. 2007;48(1):104‐113. 10.1016/j.appet.2006.07.075 16962207

