# Supplementary material for: The influence of front‐of‐pack portion size images on children's serving and intake of cereal
Source: Pediatr Obes. 2019 Nov 20;15(2):e12583. doi: 10.1111/ijpo.12583 (PMC7003796; doi:10.1111/ijpo.12583)
Supplement: Supplementary file 1 — Table S1. Results of one‐way MANCOVA showing the effects of condition (large vs small portion depiction) on three primary outcome variables (cereal serving, cereal consumption and total meal consumption), controlling for BMI, age, sex, pre‐meal ratings of liking and pre‐meal ratings of hunger. Table S2. Results of two‐way MANCOVA showing interaction effects between condition (large vs small portion depiction) and food responsiveness on the three primary outcome variables (cereal serving, cereal consumption and total meal consumption) Table S3. Results of two‐way MANCOVA showing interaction effects between condition (large vs small portion depiction) and enjoyment of food on the three primary outcome variables (cereal serving, cereal consumption and total meal consumption) [file IJPO-15-e12583-s001.docx]

**Supplementary Results**

Primary Analysis – MANCOVA Results

See main paper for justification, hypothesis and discussion of findings.

A one-way multivariate analysis of covariance (MANCOVA) was run to determine the effect of condition (large vs small portion depiction) on the three primary outcome variables (in grams): i) cereal serving, ii) cereal consumption and iii) total meal (cereal and milk) consumption, after controlling for BMI (z-score), age, sex, pre-meal ratings of cereal liking and pre-meal ratings of hunger as covariates in the model.

Overall, the model was statistically significant, indicating differences between the two portion size conditions on the combined eating behavior variables after controlling for covariates, (*F*(3,29) = 3.52, *p* = .027, Wilk's Λ = 0.73, partial η2 = .27 [see Table 1]). A significant main effect of condition (large vs small portion depiction) was found for weight of cereal served (*F*(1,31) = 5.39, *p* = .027, partial η2 =.15) and cereal consumed (*F*(1,31) = 7.16, *p* = .012, partial η2 = .19). Children in the large portion conditions both served and consumed more cereal than those shown the small portion image. However, no significant main effect of condition on the total weight of the meal (cereal and milk) consumed was found (*F*(1,31) = 3.07, *p* = .09, partial η2 = .09).

Table 1. Results of one-way MANCOVA showing the effects of condition (large vs small portion depiction) on three primary outcome variables (cereal serving, cereal consumption and total meal consumption), controlling for BMI, age, sex, pre-meal ratings of liking and pre-meal ratings of hunger.

|  | Cereal  Served (grams) | | Cereal  Consumed (grams) | | Total Meal Consumed (grams) | |
| --- | --- | --- | --- | --- | --- | --- |
| *Effect* | *F* | *p* | *F* | *p* | *F* | *p* |
| Intercept | 6.37 | .02 | .23 | .63 | .09 | .76 |
| BMI | .11 | .74 | .32 | .58 | .21 | .65 |
| Age | 2.53 | .12 | .01 | .92 | .08 | .77 |
| Sex | .65 | .43 | .43 | .52 | .24 | .63 |
| Pre-meal liking | .22 | .64 | .02 | .88 | 1.07 | .31 |
| Pre-meal hunger | .05 | .82 | .08 | .78 | .32 | .57 |
| Condition | .54 | .03 | 7.16 | .01 | 3.07 | .09 |

Note: F: MANCOVA result; MANCOVA: multivariate analysis of covariance; BMI: body mass index (age and gender adjusted z-score).

Table 2. Results of two-way MANCOVA showing interaction effects between condition (large vs small portion depiction) and food responsiveness on the three primary outcome variables (cereal serving, cereal consumption and total meal consumption)

|  | Cereal  Served (grams) | | Cereal  Consumed (grams) | | Total Meal Consumed (grams) | |
| --- | --- | --- | --- | --- | --- | --- |
| *Effect* | *F* | *p* | *F* | *p* | *F* | *p* |
| Intercept | 4.99 | .036 | .84 | .369 | .91 | .350 |
| BMI | 1.95 | .176 | .45 | .507 | .55 | .468 |
| Age | 1.97 | .173 | .73 | .403 | .59 | .450 |
| Sex | 1.73 | .202 | 1.26 | .274 | .073 | .789 |
| Pre- meal hunger | .31 | .586 | .11 | .746 | .41 | .527 |
| Pre-meal liking | .14 | .713 | .01 | .928 | .08 | .785 |
| Post-meal liking | 2.61 | .120 | 3.52 | .073 | 3.95 | .059 |
| FR | 1.85 | .186 | 2.15 | .157 | 1.03 | .321 |
| Condition | 3.85 | .062 | 9.55 | .005 | 3.01 | .096 |
| FR*Condition | 2.10 | .161 | .21 | .649 | .51 | .483 |

Note: FR: food responsiveness, FR*condition: interaction between FR and condition, BMI: body mass index [age and gender adjusted z-score].

Table 3. Results of two-way MANCOVA showing interaction effects between condition (large vs small portion depiction) and enjoyment of food on the three primary outcome variables (cereal serving, cereal consumption and total meal consumption)

|  | Cereal  Served (grams) | | Cereal  Consumed (grams) | | Total Meal Consumed (grams) | |
| --- | --- | --- | --- | --- | --- | --- |
| *Effect* | *F* | *p* | *F* | *p* | *F* | *p* |
| Intercept | 1.89 | .183 | .32 | .580 | .81 | .377 |
| BMI | 1.62 | .216 | .03 | .862 | .25 | .619 |
| Age | .62 | .438 | .88 | .358 | .87 | .362 |
| Sex | 2.26 | .146 | 1.38 | .252 | .27 | .610 |
| Pre-meal hunger | .46 | .505 | .47 | .500 | .87 | .362 |
| Pre-meal liking | .19 | .665 | .17 | .685 | .00 | .993 |
| Post-meal liking | 5.15 | .033 | 2.62 | .119 | 3.37 | .079 |
| EF | 3.40 | .058 | .21 | .652 | .04 | .846 |
| Condition | 5.18 | .032 | 7.20 | 0.13 | 2.71 | .114 |
| EF*Condition | .70 | .410 | .04 | .853 | 1.14 | .297 |

Note: FR: food responsiveness, EF*condition: interaction between EF and condition BMI: body mass index [age and gender adjusted z-score].

Secondary Analyses: CEBQ sub-scales

Food responsiveness and enjoyment of food have been shown to be associated with children’s eating and/or weight status so these individual differences in eating behaviour traits were also explored in the current study using the Child Eating Behaviour Questionnaire [CEBQ].^1–4^

Hypothesis:

Children who scored higher in enjoyment of food and food responsiveness sub-scales would have a greater intake response to the manipulation.

Results:

A series of two-way multivariate analyses of variance were run to determine whether there was an interaction between condition and the relevant Children’s Eating Behaviour Questionnaire [CEBQ] scales (food responsiveness [FR] and enjoyment of food [EF]) on the outcome variables (in grams): i) cereal serving, ii) cereal consumption and iii) total meal (cereal and milk) consumption, after controlling for BMI (z-score), age, sex, pre-meal ratings of hunger, pre-meal ratings of cereal liking and post-meal ratings of cereal liking as covariates in the model.

No significant interactions effects were found between either FR and condition [Table 2], or EF and condition [Table 3], on the combined outcome variables, (*F*(3,21) = .88, *p* = .448, partial η2 = .12 and *F*(3,21) = .92, *p* = .637, partial η2 = .08, respectively), with no significant main effects of either FR (*F*(3,21) = .79, *p* = .174, partial η2 = .21) or EF (*F*(3,21) = .84, *p* = .301, partial η2 = .16).

Discussion:

No significant differences or interactions were found for the food responsiveness and enjoyment of food scales of the CEBQ, which refer to children’s eating in response to environmental food cues.^38^ Arguably, children’s self-served portions are influenced by external, size-related cues within their environment, and so it was anticipated that children with higher scores in these scales would display a greater response to the manipulations. The use of a median split when establishing high and low scores may have resulted in a loss of sensitivity, with moderate scorers diluting any effects. This study was sufficiently powered to identify effects on serving and consumption, our primary hypotheses. However, lack of group differences may be partly due to an issue of power to adequately analyse these secondary considerations.

References

1. dos Passos DR, Gigante DP, Maciel FV, Matijasevich A. [Children’s eating behaviour: comparison between normal and overweight children from a school in Pelotas, Rio Grande do Sul, Brazil]. *Rev Paul Pediatr*. 2015;33(1):42-49. doi:10.1016/j.rpped.2014.11.007

2. Fildes A, Mallan KM, Cooke L, et al. The relationship between appetite and food preferences in British and Australian children. *Int J Behav Nutr Phys Act*. 2015;12:116. doi:10.1186/s12966-015-0275-4

3. Musher-Eizenman DR, Young KM, Laurene K, Galliger C, Hauser J, Wagner Oehlhof M. Children’s sensitivity to external food cues: how distance to serving bowl influences children’s consumption. *Health Educ Behav*. 2010;37(2):186-192. doi:10.1177/1090198109335656

4. Webber L, Hill C, Saxton J, Van Jaarsveld CHM, Wardle J. Eating behaviour and weight in children. *Int J Obes*. 2009;33(1):21-28. doi:10.1038/ijo.2008.219
